# Heterogeneity and incorporation of chromium isotopes in recent marine molluscs (*Mytilus)*

**DOI:** 10.1111/gbi.12336

**Published:** 2019-02-21

**Authors:** Sylvie Bruggmann, Robert M. Klaebe, Cora Paulukat, Robert Frei

**Affiliations:** ^1^ Department of Geoscience and Natural Resource Management, Geology Section University of Copenhagen Copenhagen Denmark; ^2^ Department of Earth Sciences University of Adelaide Adelaide South Australia Australia; ^3^ ALS Laboratory Group ALS Scandinavia AB Luleå Sweden

**Keywords:** biogenic carbonates, redox proxy, stable isotopes

## Abstract

The mollusc genus *Mytilus* is abundant in various modern marine environments and is an important substrate for palaeo‐proxy work. The redox‐sensitive chromium (Cr) isotope system is emerging as a proxy for changes in the oxidation state of the Earth's atmosphere and oceans. However, potential isotopic offsets between ambient sea water and modern biogenic carbonates have yet to be constrained. We measured Cr concentrations ([Cr]) and isotope variations (δ^53^Cr) in recent mollusc shells (*Mytilus*) from open and restricted marine environments and compared these to ambient sea water δ^53^Cr values. We found a large range in mollusc [Cr] (12–309 ppb) and δ^53^Cr values (−0.30 to +1.25‰) and in the offset between δ^53^Cr values of mollusc shells and ambient sea water ($\Delta^{53}{\text{Cr}}_{\mathrm{sea}\;\;\mathrm{water}}^{\mathrm{bulk}\;\;Mytilus}$, −0.17 to −0.91‰). Step digestions of cultivated *Mytilus edulis* specimens indicate that Cr is mainly concentrated in organic components of the shell (periostracum: 407 ppb, *n* = 2), whereas the mollusc carbonate minerals contain ≤3 ppb Cr. Analyses of individual Cr‐hosting phases (i.e., carbonate minerals and organic matrix) did not reveal significant differences in δ^53^Cr values, and thus, we suggest that Cr isotope fractionation may likely take place prior to rather than during biomineralisation of *Mytilus* shells. Heterogeneity of δ^53^Cr values in mollusc shells depends on sea water chemistry (e.g., salinity, food availability, faeces). The main control for δ^53^Cr values incorporated into shells, however, is likely vital effects (in particular shell valve closure time) since Cr can be partially or quantitatively reduced in sea water trapped between closed shell valves. The δ^53^Cr values recorded in *Mytilus* shells may thus be de‐coupled from the redox conditions of ambient sea water, introducing additional heterogeneity that needs to be better constrained before using δ^53^Cr values in mollusc shells for palaeo‐reconstructions.

## 1 INTRODUCTION

Mollusc shells are extensively used to reconstruct past sea water chemistry as they are known to record environmental parameters in their carbonate shell layers (e.g., Klein, Lohmann, & Thayer, 1996; Vander Putten, Dehairs, Keppens, & Baeyens, 2000). The genus *Mytilus* is particularly useful as it tolerates a large range of environmental conditions from high to low latitudes (Gosling, 1992). *Mytilus* are sessile and live in colonies in subtidal or intertidal zones, where they are subjected to extreme environmental conditions regarding, for example, salinity, temperature and exposure to air. Their adaptation mechanism to extreme conditions allows *Mytilus* to seal their shell valves tightly, and anoxic conditions quickly develop in the sea water trapped between the closed shell valves (Famme & Kofoed, 1980; Widdows & Shick, 1985). *Mytilus edulis* can tolerate hypoxic and anoxic sea water conditions between 5 and 16 days by closing their shell valves and decreasing oxygen consumption by utilising anaerobic metabolism to conserve energy (Babarro & Zwaan, 2008; Famme & Kofoed, 1980; Widdows & Shick, 1985; de Zwaan, 1977). As suspension feeders, *Mytilus* open their shell valves and ingest ambient sea water containing organic and inorganic particles. When low algal concentrations prevail, *Mytilus* close their shell valves (Riisgaard, Egede, & Barreiro Saavedra, 2011; Riisgaard, Lassen, & Kittner, 2006). Generally, nutrients and trace elements (e.g., ${\text{HCO}}_{3}^{-}$ , Ca^2+^, Mg^2+^, Ba^2+^, Sr^2+^) used for shell formation are derived from ingested particles or directly from sea water. These molecules are transported through two epithelial cell layers and the mantle to the extrapallial space (EPS). Crystallisation pathways of ions from the sea water to the shell via cell membranes include (a) passive ion channels (e.g., Ca^2+^, Mg^2+^, H^+^, ${\text{HCO}}_{3}^{-}$ or SO_4_^2−^ channels), (b) active ion pumps (e.g., the Ca^2+^‐pump using the enzyme Ca‐ATPase), (c) intercellular diffusion promoted by concentration gradients or (d) vacuolised fluid transport (Bentov, Brownlee, & Erez, 2009; Carré et al., 2006; Cervantes et al., 2001; Erez, 2003; Klein et al., 1996). Chromium (Cr) transport by vacuoles is supported by Chassard‐Bouchaud, Boutin, Hallegot, and Galle (1989) who observed Cr in lysosomes and vesicles of *Mytilus* cells. The authors found insoluble Cr in lysosomes associated with phosphorus and sulphur, possibly in proteins, which may prevent the toxic Cr from diffusion through the cell. In the isolated EPS, an organic matrix containing various macromolecules (e.g., polysaccharide β‐chitin, hydrophilic proteins) facilitates the formation of aragonite and calcite crystals (Addadi, Joester, Nudelman, & Weiner, 2006). An organic outer sheath consisting of different organic macromolecules (e.g., proteins, chitin; Meenakshi, Hare, Watabe, & Wilbur, 1969; Nakayama et al., 2013) protects the underlying minerals and the EPS from sea water and creates the site for calcification. The extrapallial fluid (EPF) of *M. edulis* consists of inorganic ions, insoluble sulphated carbohydrates, free amino acids and proteins, which can bind divalent cations (e.g., Ca^2+^, Cd^2+^, Mn^2+^) and potentially detoxify heavy metals (Yin, Huang, Paine, Reinhold, & Chasteen, 2015). This composition is different to the composition of the shell proteins (Misogianes & Chasteen, 1979). *Mytilus* shells secrete byssal threads, which they use to attach themselves onto a substrate. Cr was shown to be capable of adsorbing onto or complexing with the surface of byssal threads of *Mytilus* (Chassard‐Bouchaud et al., 1989).

The carbonate shell of *Mytilus* consists of two polymorphs of carbonate: an outer shell layer consisting of calcite (prismatic) and an inner shell layer of aragonite plates (nacre, columnar; e.g., Immenhauser, Schöne, Hoffmann, & Niedermayr, 2016). The morphology and nucleation process of the mineralising carbonates are dictated by an organic matrix consisting of proteins (e.g., amino acids; Dauphin, Cuif, Salomé, & Susini, 2005) and other organic macromolecules (e.g., sulphated polysaccharides; Dauphin et al., 2003). The absence of this organic matrix inhibits crystallisation of calcite and aragonite (Dauphin et al., 2003; Falini, Albeck, & Weiner, 1996).

Skeletal carbonates, such as mollusc shells, occur pervasively throughout the Phanerozoic rock record and are capable of recording the chemical composition of precipitating sea water. The Cr isotope system is increasingly used as a proxy for changes in surface redox conditions in the past as it is assumed that the delivery and distribution of Cr in sea water strongly depend on the oxygenation state of the water column (e.g., Cole et al., 2016; Ellis, Johnson, & Bullen, 2002; Frei, Gaucher, Poulton, & Canfield, 2009; Reinhard et al., 2014). With oxidative weathering of silicate rocks, Cr(III) is oxidised to soluble Cr(VI) and removed by fluids that may reach the oceans through rivers. The reductive removal of Cr(III) from these fluids to chemical sediments is consequently assumed to lead to δ^53^Cr_sea water_ values (δ^53^Cr_sea water_ ranging from 0.13 to 1.55‰; Goring‐Harford et al., 2018) that are isotopically heavier compared to bulk silicate earth (−0.12 ± 0.10‰ 2SD; Bonnand, James, Parkinson, Connelly, & Fairchild, 2013; Frei & Polat, 2013; Schoenberg, Zink, Staubwasser, & Blanckenburg, 2008). As the fractionation of Cr isotopes is generally understood to occur during redox‐sensitive reactions (Ellis et al., 2002), δ^53^Cr variations recorded in sedimentary rocks have important utility in determining changes in the redox state of ancient sedimentary basins, the oceans and Earth's evolving atmosphere. Laboratory experiments showed that Cr can directly be incorporated into the crystal lattice of carbonate minerals (Tang, Elzinga, Jae Lee, & Reeder, 2007) with insignificant or no isotope fractionation (Rodler, Sánchez‐Pastor, Fernández‐Díaz, & Frei, 2015). This renders (biogenic) carbonates as potential archives to record δ^53^Cr variations of their precipitating fluid. The offset between biogenic carbonate and ambient sea water δ^53^Cr ($\Delta^{53}{\text{Cr}}_{\mathrm{sea}\;\;\mathrm{water}}^{\mathrm{carbonate}}$ was reported in the range of 0.0–0.9‰ (Bonnand et al., 2013; Frei, Paulukat, Bruggmann, & Klaebe, 2018; Holmden, Jacobson, Sageman, & Hurtgen, 2016; Pereira, Voegelin, Paulukat, Sial, & Ferreira, 2015). Importantly, multiple analysis of a coral (*Porites* sp.) from the Rocas Atoll (BR) shows δ^53^Cr values of between −0.56 ± 0.09‰ and 0.10 ± 0.14‰ (Pereira et al., 2015) and intra‐species variations of core top planktonic foraminifera (*Pulleniatina obliquiloculata)* from the West Pacific range from 0.21 ± 0.37‰ to 1.85 ± 0.19‰ (2 *σ*; Wang et al., 2016). This heterogeneity in biogenic carbonates was attributed to changes in surface sea water δ^53^Cr values and water depth. Frei et al. (2018) and Farkaš et al. (2018) recently reported an accumulation of Cr in the carbonate shells of molluscs of the genus *Mytilus* and proposed these shells as another suitable record of sea water δ^53^Cr values. However, in contrast to Pereira et al. (2015) and Wang et al. (2016), Frei et al. (2018) report no or only little intra‐species variations in mollusc shell samples and attribute the resolvable variations to vital effects. This highlights that despite extensive investigations on biomineralisation processes of marine calcifying organisms (e.g., Bentov et al., 2009; Carré et al., 2006; Weiner & Addadi, 2011; Weiss, Tuross, & Addadi, 2002), only little is known about the transport of ions through membranes and incorporation into shells. This also applies to Cr where the transport through epithelial cells of molluscs and processes that might lead to stable metal isotope fractionation (of Cr) are not well constrained. A first model proposed by Pereira et al. (2015) for Cr uptake pathways and Cr isotope fractionation during incorporation into corals suggests that Cr(VI) is reduced by photo‐reduction and subsequently re‐oxidised during transport through the endodermal layer. After transportation through a cell membrane via vacuoles, isotopically light Cr(VI) reaches the calcifying space from where chromate can be incorporated into the coral skeleton. The uptake of Cr(VI) as chromate ions (${\text{CrO}}_{4}^{2-}$) into cells can take place in a similar way as phosphate and sulphate uptake through anionic membrane channels. This is facilitated by the structural similarity of chromate and sulphate (Brown et al., 2006). A multiple‐step intracellular reaction chain as for sulphate reduction (Canfield, 2001) was also suggested for Cr which is, similar to sulphate, first transported into the cell where the actual reduction takes place (Sikora, Johnson, & Bullen, 2008). The reduction of Cr(VI) may be important considering that Cr(VI) is toxic. Cells therefore use various mechanisms to inhibit or tolerate oxidative damage (Cervantes et al., 2001). A mechanism to inhibit damage by Cr(VI) can be the efflux of ${\text{CrO}}_{4}^{2-}$ with a specific protein (ChrA). Enzymatic or non‐enzymatic reduction of Cr(VI) to Cr(III) constitutes another mechanism to detoxify Cr(VI) (Ramírez‐Díaz et al., 2008). Thus, a transport by sulphate channels into epithelial cells and a subsequent reduction is a potential transport pathway for Cr from sea water to the EPS of molluscs. Cr(III) is not considered to permeate cell membranes (Cary, 1982) and may thus be irrelevant for Cr uptake. However, Cr(III) complexed with ligands (Cr‐L) as well as Cr(VI) contained in the body fluid or sea water can be vacuolised by the cell wall and then transported through cells. Recently, Frei et al. (2018) emphasised the importance of organic substances onto which Cr(III) is adsorbed and incorporated into mollusc shells. Here, it is important to note that complexation with organic matter (OM) may stabilise Cr(III) in solution (Sander & Koschinsky, 2000; Sander and Koschinsky, 2011) and can contribute up to 90% of total dissolved Cr in sea water (Connelly, Statham, & Knap, 2006). Also, in contrast to the typical redox‐dependent Cr isotope fractionation, redox‐independent Cr isotope fractionation occurs during complexation of Cr with ligands typically present in natural systems, such as organic acids, siderophores or Cl^‐^ (Larsen, Wielandt, Schiller, & Bizzarro, 2016; Saad, Wang, Planavsky, Reinhard, & Tang, 2017). Large amounts of OM favour the reduction of Cr(VI) to Cr(III), for example, as Cr(OH)_2_^+^, which is the stable Cr(III) species at a slightly lower pH‐Eh range of sea water (Connelly et al., 2006; Cranston & Murray, 1980).

Here, we investigate the ability of the widely abundant molluscs of the genus *Mytilus* to record δ^53^Cr values of ambient sea water and to assess their utility as a palaeo‐redox proxy archive. To this end, we propose a model for potential pathways of Cr incorporation during biomineralisation of the mollusc carbonate shell. With this model, we disentangle the pathway of Cr incorporation into the mollusc carbonate shell by investigating a series of step digestions of the organic outer sheaths (periostraca) and the shell carbonate of cultivated *M. edulis* specimens. Moreover, we discuss possible mechanisms (sea water chemistry and vital effects) that may lead to the observed variations in δ^53^Cr between sea water and carbonate shells of *M. edulis* ($\Delta^{53}{\text{Cr}}_{\mathrm{sea}\;\;\mathrm{water}}^{\mathrm{bulk}\;\;Mytilus}$).

## 2 MATERIALS AND METHODS

### 2.1 Sampling

**Figure 1:**
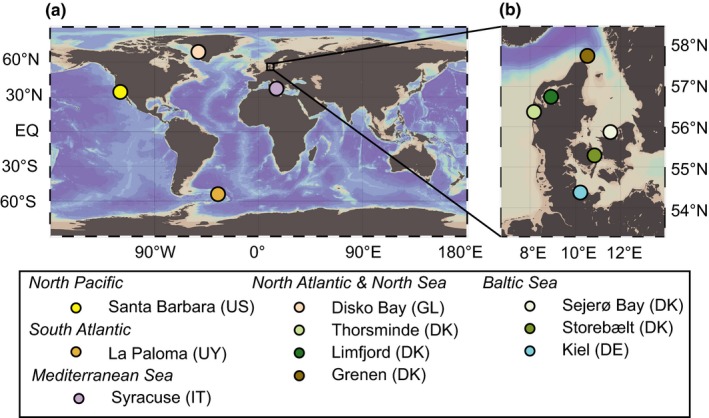
Maps (Ocean Data View, Schlitzer (2018)) indicating locations of sampled *Mytilus* (a) worldwide, (b) in Denmark and Germany [Colour figure can be viewed at wileyonlinelibrary.com]

Cultivated *M. edulis* were live‐collected (October 2016). The *M. edulis* samples grew from longlines submerged in brackish water in the Kiel Fjord (approximately 5 m water depth to sea floor; Figure 1). Ambient sea water (water temperature: 12°C, salinity: 16.5 PSU; bsh.de, https://www.bsh.de/DE/DATEN/Meeresumweltmessnetz/_Module/Stationen_mit_Frame/kiel_extern_node.html) was sampled into Nalgene © bottles. *Mytilus* from the USA (Santa Barbara, CA), Uruguay (La Paloma), Italy (Syracuse, Sicily), Greenland (Disko Bay), Denmark (various locations) and Germany (Kiel Fjord) were collected along shorelines, when possible including samples of the corresponding local surface sea water (Figure 1).

### 2.2 Cr analyses

#### 2.2.1 Sample preparation

Sea water samples (1.5–2 L) were filtered through 0.45 μm nylon membrane filters (Advantec MFS) and acidified with HCl to a pH of 2 within one day of collection. The shell tissue of all mollusc specimens collected was removed, and encrusted material was abraded using a household sponge or a ceramic knife. All shells were then pre‐washed using ultrapure Milli‐Q™ water (MQ, 18.2 MΩ cm) and 0.1 M HCl. Depending on preliminary Cr concentration analyses (by TIMS), approximately 1–3 g of single shell sides was weighed to ensure a Cr concentration of approximately 50–100 ng in the sample aliquots. These shell sample aliquots were then separated into five groups and processed differently depending on the Cr‐hosting phase(s) of interest (for the respective procedures, see Table 1). Briefly, bulk samples were incinerated and dissolved in HCl and analysed to determine the range of δ^53^Cr values in complete shells (1). The periostracum was removed from a second group of samples, dissolved in NaOCl and analysed separately (2). Similarly, Cr associated with the remaining carbonate shell (carbonate minerals and the organic matrix; L_peeled_) was analysed separately after incineration and subsequent dissolution in HCl (3). Cr associated with only carbonate minerals was separated with weak HCl (L_HCl_; 4). The organic outer sheath of multiple shell sides was leached in 7% NaOCl for different time periods of between 10 min and 72 hr (one to three individual shell sides per period of time), and the remaining shell was dissolved in HCl in order to study the effect of the periostracum on the total shell Cr (L_NaOCl_; 5). With the leaching procedure used, Cr from individual components can be separated to determine and compare the proportions of Cr in each component of interest such as the carbonate minerals and the periostracum. However, the leaching procedure used may not only leach Cr from individual components, but Cr from other components may be mixed in. Separated individual phases (carbonate minerals and periostracum) were only analysed for their [Cr], as their total Cr contents were too low for Cr isotope analysis. It is important to note that the term “separated Cr‐hosting fraction(s)” refers to phases hosting accessible Cr, that is, Cr that can be separated during ion chromatography. For example, Cr associated with organic material can only be separated by the chromatographic procedure when it was liberated from the organic material by oxidation through incineration. Despite the methodological difficulties to separate Cr associated with single components and to determine their δ^53^Cr values, mass balances were calculated using the [Cr] of theses individual phases as well as those of the bulk (1) and L_peeled_ (3) leaching steps. Every analysis of combined [Cr] and δ^53^Cr values consumes one complete shell side. Hence, the leaching procedures (Table 1) could not be conducted sequentially, but on individual components of different mollusc shells only.

**Table 1:** Sample preparation methods. Note that the separated phase(s) describe(s) only those phases, in which the hosted Cr is accessible for chromatographic Cr separation. For example, the shell organic matrix is accessible during the chromatography in L_peeled_ due to incineration, but not in L_HCl_. Organic matrix = shell organic matrix, for example, organic macromolecules. Samples were incinerated in chemical porcelain crucibles in a muffle furnace

Name	Method	Separated Cr‐hosting phase(s)
(1) Bulk	Incinerate at 750°C for 16 hr or 900°C for 2 hr^a^	Carbonate minerals, organic matrix, periostracum
	Dissolve in 6 M HCl	
(2) L_peeled_	Soak in MQ overnight to remove periostracum (peel away)	Carbonate minerals, organic matrix
	Incinerate at 750°C, 16 hr	
	Dissolve in 6 M HCl	
(3) L_HCl_	Dissolve in 0.5 M HCl overnight	Carbonate minerals
	Remove insoluble parts	
(4) L_NaOCl_	7% NaOCl for 10 min, 30 min, 2 hr, 4 hr, 16 hr, 72 hr	Carbonate minerals, organic matrix; variable amounts of periostraca (≈90%–10%)
	Rinse with MQ	
	Incinerate at 750°C, 16 hr	
	Dissolve in 6 M HCl	
(5) Periostracum	Soak in MQ overnight to peel off periostracum	Periostracum
	Dissolve in *aqua regia*	

a 750°C, 16 hr: samples from Kiel and Syracuse, 900°C, 2 hr: all other samples.

#### 2.2.2 [Cr] and stable Cr isotope analysis

All sea water samples and all acid‐digested bulk, leachate and component samples were evaporated to dryness on a hotplate and subsequently spiked with an adequate amount of a ^50^Cr‐^54^Cr double spike so that a ^50^Cr/^52^Cr ratio in the sample–spike mixture of between 0.15 and 0.70 was reached in order to minimise spike deconvolution error propagation (Ellis et al., 2002; Frei, Gaucher, Døssing, & Sial, 2011; Schoenberg et al., 2008). Next, samples were attacked with approximately 2 ml *aqua regia* to ensure equilibration between sample and spike. The sample solutions were dried down in Savillex™ Teflon beakers at 125°C overnight.

Shell samples were subjected to a two‐step ion chromatographic Cr separation modified from the protocol of D'Arcy, Babechuk, Nørbye, Gaucher, and Frei (2016). Separation of Cr from sea water samples followed the procedure described in Paulukat, Gilleaudeau, Chernyavskiy, and Frei (2016) with minor changes. Anion exchange columns were prepared with pre‐cleaned Dowex AG 1 × 8 anion resin. Samples were re‐dissolved in 20 ml (skeletal carbonates) or 200 ml (sea water) of a 0.025M HCl solution containing 0.5 ml (1 ml for sea water) of 0.2 M (NH_4_)_2_S_2_O_8_. Teflon beakers (shells) and Erlenmeyer flasks (sea water) containing the sample solutions were capped and boiled for 1 hr on a hotplate (in a sand bath for sea water samples) at 130°C to oxidise all Cr(III) to Cr(VI). Subsequently, samples cooled to room temperature were passed over the anion exchange columns, followed by rinsing with 10 ml 0.2 M HCl, 2 ml 2 M HCl, 5 ml MQ™ (25 ml 0.1 M HCl and 20 ml MQ for sea water). Cr(VI) retained in the anion resin was reduced to Cr(III) and eluted with 2 M HNO_3_ and 5% H_2_O_2_. All eluted samples were evaporated to dryness on a hotplate and re‐dissolved in 100 μl of concentrated HCl (12 M, double distilled), heated at 120°C for 10 min and diluted with 2.3 ml MQ™ water. These solutions were then passed over pre‐cleaned cation exchange columns loaded with 2 ml of a Dowex AG50W‐X8 (200–400 mesh) resin and directly collected. Cr was further collected with 8 ml of 0.5 M HCl, following a slightly modified method after Bonnand, Parkinson, James, Karjalainen, and Fehr (2011) and Trinquier, Birck, and Allègre (2008).

After Cr separation, samples were collected using 1.5 μl of a 2:1:0.5 mixture of silica gel, 0.5 M H_3_PO_4_ and 0.5 M H_3_BO_3_ and transferred onto an outgassed Re filament. [Cr] and Cr isotope analyses were conducted on an IsotopX/GV IsoProbe T thermal ionisation mass spectrometer (TIMS), where four Cr (^50^Cr^+^, ^52^Cr^+^, ^53^Cr^+^ and ^54^Cr^+^) beams were simultaneously collected together with ^49^Ti^+^, ^51^V^+^ and ^56^Fe^+^ beams to monitor potential interferences. Each sample was analysed between one and five times at ^52^Cr ion beam intensities of 300–500 mV (at approximately 1100°C). Each run consisted of 120 cycles with 10 s signal integration and included 20 s of baseline integration at ±0.5 AMU. The results were corrected for natural and instrumentally induced fractionation using the double‐spike method of Schoenberg et al. (2008) and are reported as shown in Equation (1) as δ^53^Cr in per mil (‰) with 2SD (2SE for samples with only one run).

(1) $$\delta^{53}\text{Cr}=\left(\frac{{{(}^{53}\text{Cr}{/}^{52}\text{Cr})}_{\mathrm{sample}}}{{{(}^{53}\text{Cr}{/}^{52}\text{Cr})}_{\mathrm{SRM}979}}-1\right)*1,000$$

#### 2.2.3 Data quality

Multiple analyses of double‐spiked NIST SRM 979 standard aliquots were conducted to monitor external reproducibility. The external reproducibility of the δ^53^Cr value keeping the ^52^Cr signal at 1V results in ± 0.08‰ 2SD. We measure the NIST SRM 979 standard at δ^53^Cr = −0.08‰, an offset from the certified 0‰ values that is explained by original calibration of our double spike against NIST 3112a and accounted for in all results.

Carbonate rock standards JDo‐1 and JLs‐1 were repeatedly analysed to determine the external reproducibility achieved on rock standards (Table 2), and both [Cr] and δ^53^Cr values are within the range of published values (Bonnand et al., 2011; Imai & Gloyna, 1990; Pereira et al., 2015; Rodler et al., 2015). Multiple procedural blanks (*n* = 8) remained < 3.7 ng Cr for the sea water separation procedure and ≈1 ng for bulk shell sample separation (Table 1, procedure 1). These blank concentrations are significantly below the Cr concentrations of our samples and are therefore not expected to affect the isotope composition of our samples.

**Table 2:** Carbonate standards used for external reproducibility

	[Cr] ± 2SD (ppm)	δ^53^Cr ± 2SD (‰)	*n*	Reference
JDo‐1		1.70 ± 0.08	5	Pereira et al. (2015)
	6.83 ± 0.39	1.69 ± 0.05	10	Rodler et al. (2015)
	7.26 ± 0.34	1.72 ± 0.05	10	Bonnand et al. (2011)
	7.93			Imai, Terashima, Itoh, and Ando (1996)
	6.97 ± 0.35	1.66 ± 0.08	2	This study
JLs‐1	1.14	1.80 ± 0.06	5	D'Arcy et al. (2016)
		1.90 ± 0.07	3	Pereira et al. (2015)
	1.14 ± 0.03	1.89 ± 0.02	3	Rodler et al. (2015)
	1.05 ± 0.01	1.99 ± 0.02	1	Bonnand et al. (2011)
	3.37			Imai et al. (1996)
	1.83 ± 0.19	1.95 ± 0.12	7	This study

Corrections for contamination by detrital Cr (δ^53^Cr = −0.12 ± 0.10‰) are usually conducted using concentrations of immobile elements such as Ti or Al (Algeo & Maynard, 2004; Gilleaudeau et al., 2016; Reinhard et al., 2013; Rodler et al., 2015; Schoenberg et al., 2008). The strong acids used to dissolve our samples may leach Cr associated with detrital Cr and contaminate δ^53^Cr values. We analysed aliquots of selected mollusc shell samples (bulk and L_NaOCl_; Table 1) for their Al concentrations using ICP‐MS (conducted at the Geological Survey of Denmark and Greenland, Denmark) along with a standard reference material (BHVO‐1, basalt, USGS). However, these analyses resulted in Al concentrations below detection limit (≈50 ppb), and thus, detrital Cr in our sample is not significant. This is in agreement with previous studies where Al concentrations were below detection limit (Pereira et al., 2015) and δ^53^Cr values of carbonate standards (JDo‐1 and JLs‐1) leached with 0.5 M HCl and 2 M HCl were within error (Rodler et al., 2015).

## 3 RESULTS

### 3.1 Cr in different shell components and detrital contamination

We analysed coupled bulk *M. edulis* shell samples and targeted acid‐leached samples to compare Cr in bulk shells and Cr that is associated with different components such as the shell carbonate or with OM. To rule out contamination by detrital Cr, we analysed Al of selected samples (bulk and L_NaOCl_), which resulted in Al concentrations below the detection limit (≈50 ppb). The Al concentrations measured in our samples indicate that an insignificant fraction (<1‰) of total Cr is derived from leached detrital grains and the Cr analysed is thus not thought to be associated with a detrital component.

**Figure 2:**
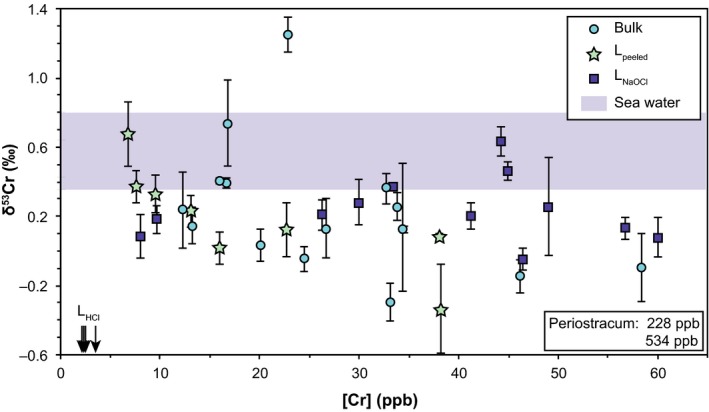
[Cr] and Cr isotope compositions of *Mytilus edulis* from Kiel Fjord (DE), with symbol shapes indicating the digestion method. The [Cr] of L_HCl_ and periostracum samples are indicated by arrows (L_HCl_) and in a box (periostraca) without δ^53^Cr values. The blue‐shaded area indicates the range of sea water δ^53^Cr values from Kiel Fjord (average [Cr] of ambient sea water is 66 ng/kg, *n* = 2) [Colour figure can be viewed at wileyonlinelibrary.com]

The step digestions allowed the determination of [Cr] and δ^53^Cr values in carbonate minerals and their associated shell organic matrices. These data are reported in Table 3 and are plotted with bulk values in Figure 2. L_peeled_ (carbonate minerals and the shell organic) show a range of [Cr] from 7 to 38 ppb and leachate samples (L_HCl_) contain Cr in concentrations of ≤ 3.4 ppb Cr. The low [Cr] of L_HCl_ samples indicates that leaching shell samples with HCl preferentially dissolved carbonate minerals and minimised leaching of organically bound Cr from the shell organic matrix. Further, the low Cr concentrations contained in L_HCl_ contribute only little or insignificantly to the total Cr concentration of the shell. The periostracum is an important host for Cr with [Cr] as high as 280–500 ppb, over two orders of magnitude higher than those in the L_HCl_ samples.

**Table 3:** [Cr] and Cr isotopic compositions of cultivated *Mytilus edulis* samples from Kiel

Digestion	Sample name	δ^53^Cr^* *^ ± 2SD^b^ (‰)	[Cr] (ppb)	$\Delta^{53}{\text{Cr}}_{\mathrm{sw}}^{x}$ (‰)
Sea water	seawater_KI‐1	0.62 ± 0.19	41.7^a^	
Sea water	seawater_KI‐2	0.50 ± 0.14	90.6^a^	
		0.56 ± 0.17	66.2^a^	
Bulk	DE‐KI_1	0.36 ± 0.09^b^	33	
Bulk	DE‐KI_3	0.74 ± 0.25	17	
Bulk	DE‐KI_4	0.26 ± 0.12	34	
Bulk	DE‐KI_19	1.25 ± 0.10^b^	23	
Bulk	DE‐KI_23	0.14 ± 0.11^b^	13	
Bulk	DE‐KI_24	0.23 ± 0.22	12	
Bulk	DE‐KI_25	0.39 ± 0.08	17	
Bulk	DE‐KI_41	0.13 ± 0.17	26	
Bulk	DE‐KI_42	−0.15 ± 0.14	46	
Bulk	DE‐KI_43	−0.30 ± 0.11^b^	33	
Bulk	DE‐KI_45	−0.10 ± 0.20	58	
Bulk	DE‐KI_48	0.13 ± 0.08	35	
Bulk	DE‐KI_49	0.40 ± 0.08	16	
Bulk	DE‐KI_78	0.14 ± 0.37	34	
Bulk	DE‐KI_80	−0.05 ± 0.08^b^	24	
Bulk	DE‐KI_85	0.03 ± 0.16	20	
Average (*n* = 16)		0.23 ± 0.74	25	−0.33
L_peeled_	DE‐KI_peeled_14	0.08 ± 0.08^b^	38	
L_peeled_	DE‐KI_peeled_33	0.33 ± 0.11^b^	10	
L_peeled_	DE‐KI_peeled_34	0.67 ± 0.18^b^	7	
L_peeled_	DE‐ KI_peeled_35	−0.34 ± 0.25^b^	38	
L_peeled_	DE‐ KI_peeled_36	0.37 ± 0.09^b^	8	
L_peeled_	DE‐ KI_peeled_37	0.01 ± 0.09^b^	16	
L_peeled_	DE‐ KI_peeled_39	0.12 ± 0.16^b^	23	
L_peeled_	DE‐ KI_peeled_40	0.23 ± 0.09^b^	13	
Average (*n* = 8)		0.19 ± 0.60	19	−0.37
L_HCl_	DE‐KI_HCl_30	n. a.	2	
L_HCl_	DE‐KI_HCl_31	n. a.	3	
L_HCl_	DE‐ KI_HCl_32	n. a.	2	
Average (*n* = 3)			2	
L_NaOCl_ (0.17)	DE‐ KI_NaOCl_58	0.20 ± 0.09^b^	26	
L_NaOCl_ (0.5)	DE‐KI_NaOCl_51	0.28 ± 0.09	30	
L_NaOCl_ (0.5)	DE‐ KI_NaOCl_52	0.46 ± 0.40	45	
L_NaOCl_ (0.5)	DE‐ KI_NaOCl_53	0.26 ± 0.16	49	
Average (0.5) (*n* = 3)		0.33 ± 0.12	41	−0.23
L_NaOCl_ (2)	DE‐ KI_NaOCl_60	0.13 ± 0.11	57	
L_NaOCl_ (2)	DE‐ KI_NaOCl_66	0.63 ± 0.08	44	
L_NaOCl_ (4)	DE‐ KI_NaOCl_68	0.37 ± 0.09	33	
L_NaOCl_ (16)	DE‐ KI_NaOCl_54	0.08 ± 0.12	60	
L_NaOCl_ (16)	DE‐ KI_NaOCl_55	0.20 ± 0.09^b^	41	
L_NaOCl_ (16)	DE‐ KI_NaOCl_56	−0.05 ± 0.08^b^	46	
L_NaOCl_ (72)	DE‐ KI_NaOCl_64	0.18 ± 0.12^b^	10	
L_NaOCl_ (72)	DE‐ KI_NaOCl_65	0.08 ± 0.08	8	
Average (72) (*n* = 2)		0.13 ± 0.04	9	−0.43
Periostracum	DE‐ KI_perio_69	n. a.	534	
Periostracum	DE‐ KI_perio_70	n. a.	280	
Average (*n* = 2)		0.37^c^	407	−0.19

The number of samples is indicated by *n*.

a [Cr] of sea water samples are in ng/kg.

b 2SE, n.a. = no analysis.

c Calculated after Equation (2), sw = sea water, *x* can be substituted by bulk, L_peeled_, L_HCl_, L_NaOCl_ or periostraca.

Cr contained in carbonate minerals is, together with Cr associated with the organic matrix, included in L_peeled_ samples (*m*_peeled_ = *m*_total_ − *m*_periostracum_ ≈ 2.17 g). L_peeled_ samples contain average [Cr] of 19 ppb, which is 75% of total Cr. Despite the small mass fraction of periostraca (1.4 wt%) relative to the total mass of the shells (*m*_total_ ≈ 2.2 g and *m*_periostracum_ ≈ 0.03 g), our calculations show that on average ([Cr]  = 407 ppb), periostraca contribute with 22% to the total Cr of a *Mytilus* shell. Average [Cr] contained in L_peeled_ (19 ppb; 75% of total Cr) and in periostraca (407 ppb; 22% of total Cr) add up to approximately 97% of Cr analysed in bulk *M. edulis* shells (25 ppb).

**Figure 3:**
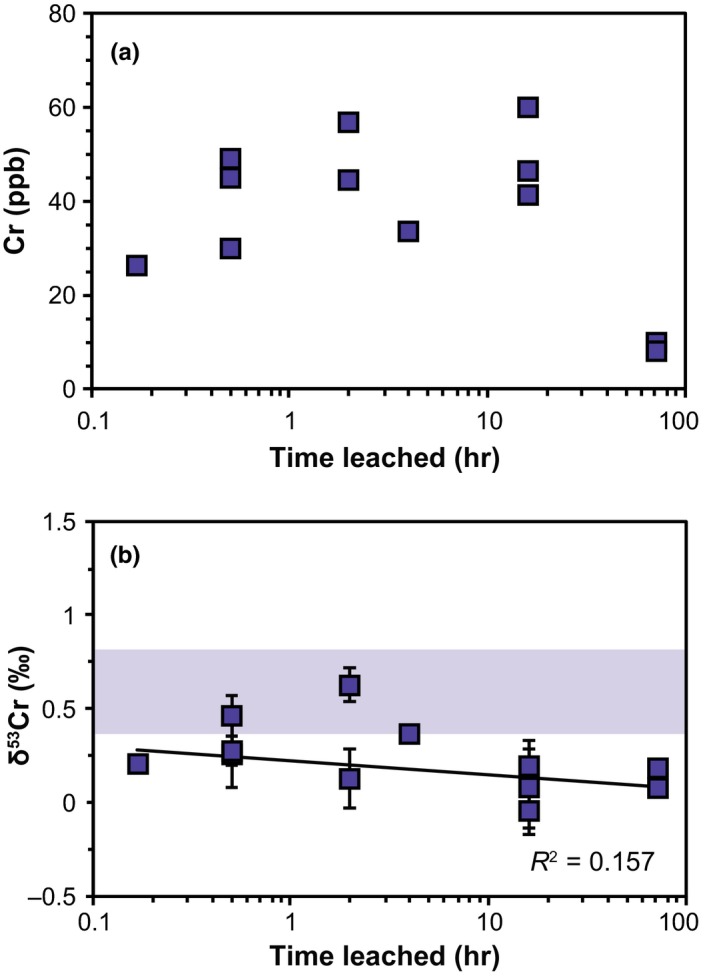
Individual shell sides of *Mytilus edulis* from Kiel Fjord were leached in 7% NaOCl for different time increments (L_NaOCl_). (a) [Cr], (b) Cr isotopic compositions; blue‐shaded area: ambient δ^53^Cr_sea water_ values [Colour figure can be viewed at wileyonlinelibrary.com]

Analyses of combined carbonate minerals and shell organic matrix (L_peeled_) reveal heterogeneous δ^53^Cr values of between −0.34 ± 0.25‰ and 0.67 ± 0.18‰. The total Cr content of both L_HCl_ samples and periostraca is too low (approximately 9 ng Cr) to analyse for their δ^53^Cr values. To characterise variations in Cr concentrations and δ^53^Cr values between samples with nearly complete periostraca and samples without periostraca, we leached individual shell sides with NaOCl for different time periods (L_NaOCl_; Figure 3). Since it was not possible to step leach the same shell specimen and analyse a time‐series of the Cr isotopic composition of all fractions, the results of the leached shells are susceptible to variations in [Cr] and δ^53^Cr values. Our analyses of L_NaOCl_ do not show a change in [Cr] for short (up to 16 hr) leaching periods. Only after 72‐hr leaching time, [Cr] fall significantly to 8–10 ppb from previously 26–60 ppb (leached with NaOCl for up to 16 hr) (Table 3; Figure 3). Samples containing the complete (bulk: 0.23 ± 0.74‰ 2SD, *n* = 16) or nearly intact (L_NaOCl, 0.5 hr_: 0.33 ± 0.12‰, *n* = 3) periostraca are isotopically heavier than samples where the periostraca were removed (L_peeled_: 0.19 ± 0.60‰, *n* = 8; L_NaOCl, 72 hr_: 0.13 ± 0.04‰, *n* = 2; Figures 2 and 3; Table 3). However, NaOCl leachates (L_NaOCl_) likely overestimate the offset since NaOCl leaches not only the periostracum, but to some extent also Cr associated with the shell organic matrix. To determine the δ^53^Cr value of periostraca of *M. edulis*, we calculated an approximate δ^53^Cr_periostracum_ value using a mass balance equation (Equation (2)) and average values for Cr concentrations and isotopic values of bulk samples ([Cr]_bulk_, δ^53^Cr_bulk_., L_peeled_ samples ([Cr]_Lpeeled_, δ^53^Cr_Lpeeled_) and carbonates ([Cr]_LHCl_).

(2) $$\delta^{53}{\text{Cr}}_{\mathrm{periostracum}}=\frac{\delta^{53}{\text{Cr}}_{\mathrm{Lpeeled}}*{\left[\text{Cr}\right]}_{L_{\mathrm{HCl}}}-\delta^{53}{\text{Cr}}_{\mathrm{bulk}}*{\left[\text{Cr}\right]}_{\mathrm{bulk}}}{{\left[\text{Cr}\right]}_{\mathrm{Lpeeled}}-{\left[\text{Cr}\right]}_{\mathrm{bulk}}}$$

The mass balance equation results in a δ^53^Cr_periostracum_ value of 0.37‰ (Table 3). The average values used to calculate the δ^53^Cr_periostracum_ value are not optimally representing the distinct Cr pools, and thus, the result of the mass balance calculation is only an approximation to the Cr isotopic composition of the periostracum.

### 3.2 Cr in bulk *Mytilus* samples

**Figure 4:**
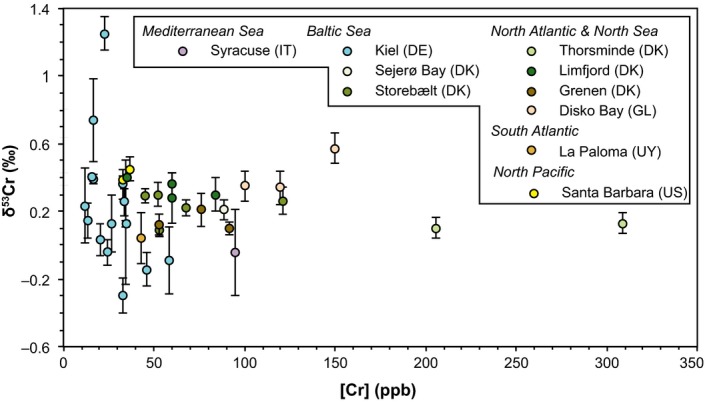
Bulk [Cr] and δ^53^Cr values of *Mytilus* samples from different sampling sites with different colours indicating different locations [Colour figure can be viewed at wileyonlinelibrary.com]

In addition, bulk *Mytilus* shells and ambient sea water from different locations were measured to better constrain spatial (global) Cr variability as well as the range of variability at individual locations. The results of bulk analysis of *Mytilus* shells from 10 locations sampling different ocean basins show spatial variations as well as some location‐specific heterogeneity in [Cr] and δ^53^Cr values (Figures 1 and 4; Table 4). The [Cr] span a large range of between 12 and 309 ppb. The relatively large number of samples (*n* = 16) from Kiel Fjord reveals a range for bulk shell [Cr] between 12 and 58 ppb. *Mytilus* shells from Disko Bay show a comparable range of [Cr] of individual shell samples (between 100 and 150 ppb). The largest variability in [Cr] ranges from 206 to 309 ppb and was found in *Mytilus* shells from Thorsminde (DK).

**Table 4:** List of all analysed samples with δ^53^Cr_sw_ and $\Delta^{53}{\text{Cr}}_{\mathrm{sw}}^{\mathrm{bulk}}$ (sw = sea water) values indicated where available for average δ^53^Cr_bulk_ values

Location	Digestion	Sample name	Species	δ^53^Cr_*Mytilus*_ ± 2SD^a^ (‰)	[Cr] (ppb)	δ^53^Cr_sw_ ± 2SD (‰)	$\Delta^{53}{\text{Cr}}_{\mathrm{sw}}^{\mathrm{bulk}}$ (‰)
North Pacific							
Santa Barbara (CA), United States	Sea water					0.77 ± 0.08^b^	0.35
	Bulk	STBA‐1	*Mytilus californianus*	0.45 ± 0.07	37		
	Bulk	STBA‐2	*M. californianus*	0.39 ± 0.02	33		
	Average			0.42 ± 0.08	35		
North Atlantic and North Sea							
Thorsminde, DK	Sea water					1.02 ± 0.08^b^	0.91
	Bulk	DK‐TM1	*Mytilus edulis*	0.10 ± 0.06	206		
	Bulk	DK‐TM2	*M. edulis*	0.13 ± 0.06	309		
	Average			0.12 ± 0.04	258		
Grenen (Skagerrak), DK	Sea water					1.00 ± 0.08^b^	0.86
	Bulk	DK‐GS1	*M. edulis*	0.21 ± 0.10	76		
	Bulk	DK‐GS2	*M. edulis*	0.12 ± 0.06	53		
	Bulk	DK‐GS3	*M. edulis*	0.10 ± 0.04	92		
	Average			0.14 ± 0.12	74		
Limfjord, DK	Bulk	DK‐LF1	*M. edulis*	0.40 ± 0.02	35		
	Bulk	DK‐LF2	*M. edulis*	0.30 ± 0.1	84		
	Bulk	DK‐LF3	*M. edulis*	0.28 ± 0.15	60		
	Bulk	DK‐LF4	*M. edulis*	0.36 ± 0.08	60		
	Average			0.34 ± 0.11	60		
Disko Bay, GL	Sea water					0.73 ± 0.10^b^	0.31
	Bulk	GL‐DB1	*M. edulis*	0.35 ± 0.09	100		
	Bulk	GL‐DB2	*M. edulis*	0.34 ± 0.1	120		
	Bulk	GL‐DB3	*M. edulis*	0.57 ± 0.09	150		
	Average			0.42 ± 0.26	123		
South Atlantic							
La Paloma, UY	Bulk	Uy‐LP1	*Mytilus* sp.	0.04 ± 0.15	43		
Mediterranean Sea							
Syracuse (Sicily), IT	Bulk	IT‐SI1	*M. edulis*	−0.04 ± 0.26^a^	95		
Baltic Sea, Sound, Kattegat							
Sejerø Bay, DK	Bulk	DK‐SO1	*M. edulis*	0.21 ± 0.06	89		
Storebælt (Funen), DK	Sea water					0.40 ± 0.02^b^	0.17
	Bulk	DK‐Fyn1	*M. edulis*	0.09 ± 0.04	53		
	Bulk	DK‐Fyn2	*M. edulis*	0.30 ± 0.07	52		
	Bulk	DK‐Fyn3	*M. edulis*	0.22 ± 0.05	68		
	Bulk	DK‐Fyn4	*M. edulis*	0.29 ± 0.03	45		
	Bulk	DK‐Fyn5	*M. edulis*	0.26 ± 0.06	121		
	Average			0.23 ± 0.17	68		

a 2SE.

b Data from Paulukat et al. (2016).

Both the minimum (0.30 ± 0.11‰) and maximum (+1.25 ± 0.10‰ 2SD) bulk δ^53^Cr values of all shell samples measured in this study are found in shells from Kiel Fjord (Figure 4 and Table 3). Excluding shell samples from Kiel Fjord, the δ^53^Cr values of shell samples from all other locations range from −0.04 ± 0.26‰ to 0.57 ± 0.09‰. The δ^53^Cr values of individual shell samples from Storebælt vary between 0.09 ± 0.04‰ and 0.30 ± 0.07‰ and from Disko Bay between 0.34 ± 0.01‰ and 0.57 ± 0.09‰. Storebælt (Funen, Denmark) and Sejerø Bay (Zealand, Denmark; Table 4, Figures 1 and 4) record similar average δ^53^Cr values: δ^53^Cr_Storebælt_ = 0.23 ± 0.17‰, δ^53^Cr_Sejerø Bay_ = 0.21 ± 0.08‰, δ^53^Cr_Kiel_ = 0.23 ± 0.74‰; average δ^53^Cr_Baltic inland sea_ = 0.22 ± 0.02‰). These samples from Baltic Sea locations differ from *Mytilus* from the U.S. West Coast (δ^53^Cr_Santa Barbara_ = 0.42 ± 0.08‰), from Uruguay (δ^53^Cr_La Paloma_ = 0.04 ± 0.15‰) and from Greenland (δ^53^Cr_Disko Bay_ = 0.42 ± 26‰).

### 3.3 Isotopic offset between bulk *Mytilus* and sea water

**Figure 5:**
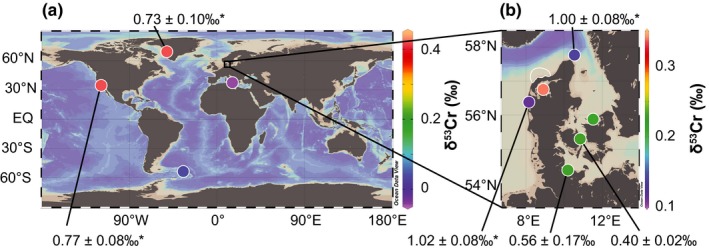
Maps (Ocean Data View, Schlitzer (2018)) indicating average δ^53^Cr values of *Mytilus* (colour‐coded circles with colour bars on the left of plots indicating δ^53^Cr values) collected at different locations: (a) global map with samples from North and South Atlantic, North Pacific, Mediterranean Sea; (b) map representing samples from North Sea and Baltic Sea. Numbers (‰) indicate δ^53^Cr_sea water_ values of different locations (also see Tables 3 and 4). * indicate δ^53^Cr_sea water_ values from Paulukat et al. (2016) [Colour figure can be viewed at wileyonlinelibrary.com]

All *Mytilus* samples (averages) are generally characterised by less positive Cr isotopic compositions relative to local sea water (Tables 3 and 4; Figures 4 and 5). The $\Delta^{53}{\text{Cr}}_{\mathrm{sea}\;\;\mathrm{water}}^{\mathrm{bulk}\;\;Mytilus}$ offsets of (average) *Mytilus* vary between 0.17‰ and 0.91‰ across all locations (Table 4). At Kiel Fjord, two sea water samples were taken during the shell sampling and return a range of δ^53^Cr values between 0.62 ± 0.19‰ and 0.50 ± 0.14‰ (Table 3). These two samples define an average δ^53^Cr value of 0.56 ± 0.17‰ which is within the range of published values for surface sea water from the Baltic Sea (0.13 ± 0.06‰ to 0.63 ± 0.13‰, Paulukat et al., 2016). These bulk values from cultivated *M. edulis* samples result in an average offset of bulk shells from local surface sea water of $\Delta^{53}{\text{Cr}}_{\mathrm{sea}\;\;\mathrm{water}}^{\mathrm{bulk}\;\;Mytilus}$ = 0.33‰ (*n* = 16).

## 4 DISCUSSION

Our results of step digestions of cultivated *M. edulis* (Kiel Fjord, DE) provide crucial information on Cr‐hosting phases in carbonate shells contributing to our understanding of Cr uptake mechanisms into biogenic carbonates. This approach helps to characterise potential impact of vital effects (e.g., feeding strategies, calcification mechanism) on δ^53^Cr values in bulk *M. edulis* shells and their offset from sea water ($\Delta^{53}{\text{Cr}}_{\mathrm{sea}\;\;\mathrm{water}}^{\mathrm{bulk}\;\;Mytilus}$). The heterogeneity of both [Cr] and δ^53^Cr values in *Mytilus* between different locations may indicate locally or globally variable sea water chemistry influencing the δ^53^Cr values of precipitating carbonates and their isotopic offset from sea water.

### 4.1 Cr concentration and isotope variability during incorporation into cultivated *M. edulis* (Kiel Fjord)

The heterogeneity of δ^53^Cr values in *M. edulis* from Kiel Fjord (Figure 1) may be caused by varying amounts of Cr‐hosting components with distinct δ^53^Cr values. The calculated δ^53^Cr value of a periostracum of a *Mytilus* shell (0.37‰) is higher than other shell fractions and can contain large amounts of Cr. Periostraca are easily abraded, and variable amounts of periostraca of individual shells can potentially influence their bulk δ^53^Cr value and thus the heterogeneity in δ^53^Cr values from Kiel Fjord (−0.30 ± 0.11‰ to 1.25 ± 0.10‰). Furthermore, periostraca may be susceptible to short‐term changes in δ^53^Cr_sea water_ since they can adsorb Cr directly from sea water similarly to byssal threads (Chassard‐Bouchaud et al., 1989). However, also the shell samples without periostraca (L_peeled_) exhibit a large range of δ^53^Cr values (−0.34 ± 0.25‰ to 0.67 ± 0.18‰; Figure 2), similar to the one of bulk shells. These shell fractions (L_peeled_) contain Cr from both the organic matrix and carbonate minerals. Since L_HCl_ samples contribute only little Cr ([Cr] of between 2 and 3 ppb; 13%), we propose that large proportions of Cr are likely incorporated into the shell valves in association with the shell organic matrix and the periostracum. This contrasts with previous studies suggesting that Cr is incorporated into the carbonate minerals of corals or of experimentally precipitated calcite (Pereira et al., 2015; Rodler et al., 2015; Tang et al., 2007). The δ^53^Cr variability in *M. edulis* from Kiel Fjord may be incorporated in the organic matrix of the shell rather than resulting from different amounts of periostraca.

#### 4.1.1 Cr fractionation factors in cultivated *M. edulis*

Assessing potential Cr isotope fractionation mechanisms that might take place both prior to and during Cr uptake may help to understand the heterogeneity in δ^53^Cr values in *M. edulis* from Kiel Fjord. Having identified the shell fractions containing Cr (mainly periostraca and shell organic matrix) in *M. edulis* from Kiel Fjord, we calculate Cr fractionation factors (ε*) that correlate the δ^53^Cr value of the product (shell) with the reactant (sea water) to characterise possible Cr uptake pathways. The calculated fractionation factors (ε*) are based on ε (Equation (3)).

(3) $$\varepsilon =\delta^{53}{\text{Cr}}_{\mathrm{product}}-\delta^{53}\;{\text{Cr}}_{\mathrm{reactant}}$$

Using a Rayleigh fractionation model, the transient Cr isotopic composition (δ^53^Cr_t_) is calculated by setting the initial Cr isotopic composition (δ^53^Cr_0_) in relation to the “remaining” Cr fraction (*f*) while removing/adding Cr with a constant fractionation factor (α) (Equation (4); Rayleigh, 1896). The Cr fraction (*f*) is calculated as the ratio of the transient [Cr] (C_t_) and the initial [Cr] (C_0_; Equation (5)). Since the initial Cr reservoir (C_0_) shows lower [Cr] than the product (shell), Cr_t_ was increased from a starting point of [Cr]_sea water_ to [Cr]_shell_.

(4) $$\delta^{53}{\text{Cr}}_{t}=\left(\delta^{53}{\text{Cr}}_{0}+1,000\right)*f^{\left(\alpha -1\right)}-1,000$$

(5) $$f=\frac{C_{t}}{C_{0}}$$

The fractionation factor α characterises the ^53^Cr/^52^Cr ratios of the product and the reactant (Equation (6)), and its association with the fractionation factor ε* is described in Equation (1).

(6) $$\alpha =\frac{{{(}^{53}\text{Cr}{/}^{52}\text{Cr})}_{\mathrm{product}}}{{{(}^{53}\text{Cr}{/}^{52}\text{Cr})}_{\mathrm{reactant}}}$$

(7) ε=1,000∗(α−1)

**Figure 6:**
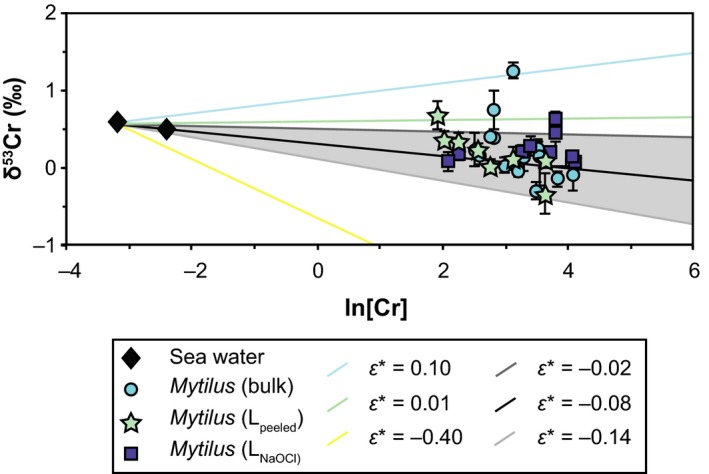
Ln[Cr] ([Cr] in ppb) and δ^53^Cr values of *Mytilus edulis* from Kiel Fjord with different digestion methods indicated by symbol shapes; ambient sea water samples are represented by black diamonds. Lines indicate fractionation factors (ε*; *f* ≥ 1) of Cr reduction (yellow; Han et al., 2012) and Cr oxidation (green and blue; Zink et al., 2010; Bonnand et al., 2013), and black and grey lines (highlighted by grey‐shaded area) indicate ε* values that best represent the Cr fractionation of most *M. edulis* samples from sea water [Colour figure can be viewed at wileyonlinelibrary.com]

We note that ε* uses *f *≥* *1 and thus differs from ε. Fractionation factors for both Cr reduction (−0.38‰; Han, Qin, Brown, Christensen, & Beller, 2012) and oxidation (≈+0.2‰; Zink, Schoenberg, & Staubwasser, 2010) from published literature were used to approach a fractionation factor ε* suitable for Cr incorporation into *Mytilus*. The majority of cultivated *M. edulis* samples (Kiel Fjord, DE) regardless of the digestion method fall in a range of fractionation factors of between ε* = −0.14‰ and ε* = −0.02‰ (Figure 6). Compared with the large variations in fractionation factors caused by Cr reduction and oxidation, the identified fractionation factors ε* are only slightly lower than the ε suggested for fractionation between sea water and (inorganically) precipitated carbonate (<0.2‰; Bonnand et al., 2013). Individual Cr‐hosting phases do not have distinct and/or consistent δ^53^Cr signatures and specific fractionation factors. Variations in bulk δ^53^Cr values of *M. edulis* specimens from a single cultivation site cannot be caused by different amounts of Cr‐hosting fractions since these fractions do not show distinct ε* or δ^53^Cr values (e.g., shell organic matrix). Instead, this indicates that the highly variable ratios of ^53^Cr/^52^Cr are incorporated into the shell, likely into the shell organic matrix. Thus, both Cr(III) and Cr(VI) can be transported to the EPS and be incorporated into *M. edulis* shells.

#### 4.1.2 Cr incorporation model into cultivated *M. edulis*

The δ^53^Cr values of the majority of our bulk, L_peeled_ and L_NaOCl_ *M. edulis* samples from Kiel Fjord (DE) are lower than ambient sea water δ^53^Cr values with fractionation factors between ε* −0.14‰ and −0.02‰ (Figure 2 and Table 3). This implies that reduced and isotopically light Cr(III) seems to be the preferential Cr isotope transported through epithelial cells to the calcifying space of *Mytilus* and from there incorporated into the shell organic matrix and associated carbonated minerals (L_peeled_). This is in agreement with other studies identifying δ^53^Cr_carbonate_ values lower than δ^53^Cr_sea water_ values and supports a reductive uptake pathway for Cr (Bonnand et al., 2013; Farkaš et al., 2018; Frei et al., 2018; Holmden et al., 2016; Pereira et al., 2015; Wang et al., 2016). Nevertheless, some bulk samples and shell fractions of *M. edulis* show δ^53^Cr values similar or higher than sea water and require a Cr transport mechanism that is capable of transporting isotopically heavy Cr(VI).

**Figure 7:**
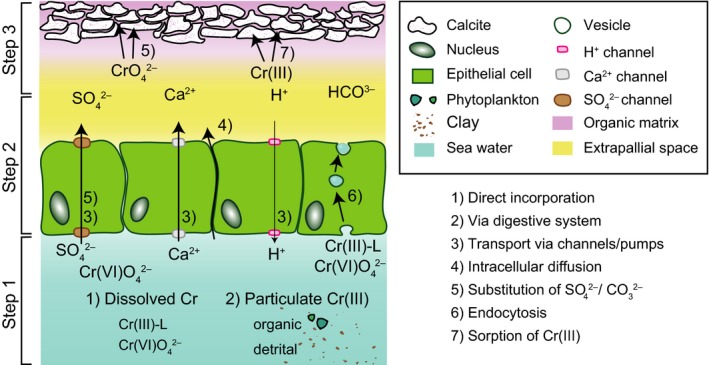
Model for Cr transport pathways of different ions from sea water through epithelial cells to the extrapallial space (EPS) and eventually into the shell organic matrix/carbonate crystals. Possible processes involved are indicated by numbers and described in the lower left corner. Only one of two layers of epithelial cells is illustrated for simplification. Model after, for example, Cervantes et al. (2001), Bentov et al. (2009), and Joutey, Sayel, Bahafid, and El Ghachtouli (2015) [Colour figure can be viewed at wileyonlinelibrary.com]

Based on the understanding of Cr transport through (microbial) cells and our results from Cr analyses of *M. edulis* from Kiel Fjord (DE), we discuss a three‐step model for Cr(III) and Cr(VI) uptake. Figure 7 shows a simplified illustration of the three‐step pathway from sea water through one layer of epithelial cells and into the EPS where an inner calcitic shell layer is precipitated with interaction with molecules of the organic matrix. (a) A mollusc shell first takes up Cr into its body cavity. (b) Cr is then transported through epithelial cells to the EPS and (c) subsequently incorporated into the shell valves.

Sea water contains dissolved Cr as chromate (${\text{CrO}}_{4}^{2-}$) or complexed with ligands (Cr‐L) as well as particle‐bound Cr, that is, Cr adsorbed to and/or structurally incorporated into detrital (e.g., silt or clay) and organic (e.g., phytoplankton) particles. *Mytilus* filter sea water for feeding and are able to open and close their shell valves depending on food availability (step 1). While particle‐bound Cr is ingested into the digestive system, dissolved Cr species can directly be accumulated from sea water. To reach the EPS from the body cavity or the digestive system, Cr has to pass two layers of epithelial cells (step 2). As a possible transport pathway of Cr through an epithelial cell, dissolved chromate may be transported via sulphate channels (Figure 7, reaction 5). This was observed for microbial cells where chromate can enter cells via sulphate channels and is effluxed by the ChrA protein (Cervantes et al., 2001). In another possible pathway, Cr contained in sea water or the body fluid is vacuolised by an epithelial cell via endocytosis (Bentov & Erez, 2006). Cr is then transported through the cell, possibly as Cr(III) in association with sulphur and phosphorus (Chassard‐Bouchaud et al., 1989), before it is exocytosed into the EPS (Figure 7, reaction 6). From the EPS, chromate may be bound to the organic matrix where it may substitute organic sulphates (e.g., acidic sulphated sugars; Dauphin et al., 2003, 2005) that are major constituents of the organic matrix (step 3). In the EPF, Cr(III) could form new chelates with available ligands, similar to Mn^2+^ (Misogianes & Chasteen, 1979). Cr(III) adsorbed onto organic macromolecules was proposed to bind to chitin‐containing organic interlamellar sheets of the organic matrix (Frei et al., 2018; Nudelman et al., 2008; Suzuki & Nagasawa, 2013). The low proportions of Cr associated with the carbonate minerals (approximately 13% in L_HCl_) suggest that the quantities of Cr incorporated into the crystal lattice during calcification of molluscs may be very small. This contrasts the findings of Pereira et al. (2015) who suggested Cr(VI) incorporation into the lattice of carbonate crystals in corals. However, the growth mechanisms of corals are considerably different from the mechanisms used by molluscs since coral growth involves symbiotic algae (zooxanthellae).

#### 4.1.3 Cr fractionation during sea water ingestion and Cr uptake

**Figure 8:**
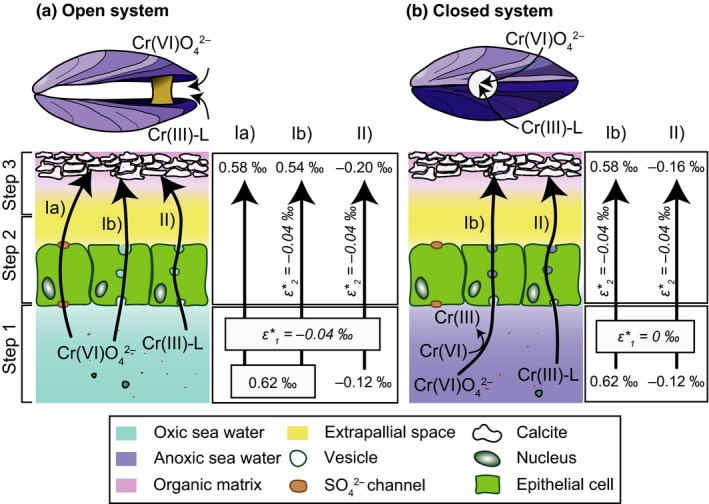
Conceptual model and box model illustrating Cr uptake from sea water under open (a) or closed system conditions (b). Two cases are considered, where nearly 100% of total Cr is present I) as Cr(VI) with a δ^53^Cr_sea water, maximum_ of 0.62 ± 0.19‰ and II) as Cr(III) with a δ^53^Cr_sea water_ of approximately the bulk silicate earth value (−0.12 ± 0.10‰; Schoenberg et al., 2008). We propose two possible sites for Cr fractionation, step 1 with $\varepsilon_{1}^{*}$ and step 2 with $\varepsilon_{2}^{*}$. (a) Under open system conditions with normal valve gaping rates, Cr(VI) is reduced to Cr(III) in step 1 with a fractionation factor of $\varepsilon_{1}^{*}$ = −0.04, regardless of the dominant Cr species. In step 2, Cr(VI) can be transported through epithelial cells via sulphate channels without fractionation, resulting in a δ^53^Cr_*M. dulis*_ value close to sea water (Ia: +0.58‰). Further, C(VI) transport via vesicles could induce Cr reduction with $\varepsilon_{2}^{*}$ = −0.04‰, leading to a δ^53^Cr_*M. edulis*_ value of +0.54‰ (Ib). If Cr(III) is the dominant Cr species, the small proportion of Cr(VI) present could be reduced during vesicular transport ($\varepsilon_{2}^{*}$ = −0.04‰), leading to a low δ^53^Cr_*M. edulis*_ value of −0.20‰ (II: δ^53^Cr_*M. edulis*_ < δ^53^Cr_sea water_). (b) In a closed system with long‐term closed shell valves, sea water trapped between the valves becomes anoxic, leading to quantitative Cr reduction (step 1, $\varepsilon_{1}^{*}$ = 0‰). Reduced Cr(III) is then transported via vesicles to the EPS, assuming a fractionation factor ($\varepsilon_{2}^{*}$) of −0.04‰ for both Cr(VI)‐ and Cr(III)‐dominated systems. The δ^53^Cr_*M. edulis*_ values could thus result in +0.58‰ (Ib) and −0.16‰ (II). Model after, for example, Cervantes et al. (2001), Bentov et al. (2009), and Joutey et al. (2015) [Colour figure can be viewed at wileyonlinelibrary.com]

The physiology of *Mytilus* provides ideal conditions to cause a redox‐dependent Cr fractionation because anoxic conditions can quickly evolve between closed shell valves due to the immediate drop of oxygen uptake from 0.2 ml O_2_ g^−1^ hr^−1^ to 0 ml O_2_ g^−1^ hr^−1^ (Famme & Kofoed, 1980; Widdows & Shick, 1985). We hypothesise that Cr fractionation with variable fractionation factors likely takes place in sea water trapped between the shell valves due to changing redox conditions (step 1; Figure 8). Since the epithelial cells are protected from environmental changes and the conditions within cells are thought to be constant, possible fractionation during cellular transport (step 2) is assumed to be constant and small.

The box models in Figure 8 show basic calculations for (a) open and (b) closed system conditions, referring to open and closed shell valves. The mean ε* value (−0.08‰) that was previously calculated for *M. edulis* (bulk and individual shell components) from Kiel Fjord was used as total Cr isotope fractionation ($\varepsilon_{1}^{*}$ + $\varepsilon_{2}^{*}$). To simplify the calculations, both step 1 and step 2 of the model are assumed to equally contribute to the total Cr isotope fractionation (if $\varepsilon_{1}^{*}$ ≠ 0‰: $\varepsilon_{1}^{*}$ = $\varepsilon_{2}^{*}$ = −0.04‰). The first scenario (I) illustrates a Cr(VI)‐dominated scenario (nearly 100% Cr(VI) with the maximum δ^53^Cr_sea water_ measured in Kiel Fjord (0.62 ± 0.19‰). Cr(VI) is transported to the EPS Ia) via sulphate channels or Ib) via vesicular transport. A second scenario (II) is dominated by Cr(III) (nearly 100% Cr(III), assuming a δ^53^Cr value of bulk silicate earth (−0.12 ± 0.10‰ (2SD); Schoenberg et al., 2008)). Cr(III) is transported through the cells with vesicles. We emphasise that these calculations are based on theoretical values and only provide an approximation of natural Cr isotope values.

Depending on the period of shell valve closure time (average closure time 59 ± 22 min; Riisgaard et al., 2006), Cr(VI) will be partially or quantitatively reduced. Under open system conditions with normal food availability and thus normal valve gaping rate, Cr may be partially reduced (Figure 8a) due to the onset of anoxic conditions (step 1). Regardless whether the sea water is Cr(VI)‐ or Cr(III) dominated, Cr(VI) that is trapped between the valves can be partially reduced with an $\varepsilon_{1}^{*}$ of −0.04‰. Subsequently, Cr is transported through epithelial cells, a process that also might induce Cr isotope fractionation. Although we cannot rule out Cr isotope fractionation occurring during transport through sulphate channels, we do not suspect any fractionation as no redox changes take place (Figure 8a). The transported Cr(VI) may then directly be fixed in the carbonate shell without inducing Cr isotope fractionation (step 3). Hence, this pathway (Ia, Figure 8a) could lead to relatively high sea water‐like δ^53^Cr values in open systems (e.g., 0.58‰), as observed in some bulk *Mytilus* shells. As an alternative transport pathway, both Cr(VI) (Ib) and Cr(III) (II) can be transported through cells via vesicles. However, it is unclear in which speciation Cr is present in vesicles. A reduction of Cr(III) within the vesicles ($\varepsilon_{2}^{*}$ = −0.04‰) could contribute to explaining the generally negative Cr isotopic offset between sea water and *Mytilus* shells (Ib) 0.54‰ and II) −0.20‰).

Closed system conditions where the shell valves are sealed during longer time periods of up to 12.2 days occur during starvation periods or due to exposure to air (Babarro & Zwaan, 2008). In such a closed system with anoxic conditions, quantitative reduction with $\varepsilon_{1}^{*}$ = 0‰ can take place in both Cr(VI)‐ and Cr(III)‐dominated sea water trapped between shell valves (Figure 8b, step 1). Both abiotic and biotic Cr reduction reactions are fast enough to be able to reduce a significant fraction of Cr(VI) in the entrapped fluid to Cr(III), making it adsorptive to organic molecules or reactive towards organic ligands present in the trapped sea water. Abiotic reduction reactions as, for example, reactions with Fe(II)‐bearing minerals can reduce 50% of total Cr(VI) within 35 min while biotic reduction requires 4 hr to 2 days (Basu and Johnson, 2012; Sikora et al., 2008). Vesicular Cr transport through epithelial cells may also induce Cr isotope fractionation ($\varepsilon_{2}^{*}$ = −0.04‰). The resulting δ^53^Cr_*M. edulis*_ values may range between 0.58‰ (Ib) and −0.16‰ (II). Thus, different Cr‐speciation scenarios coupled with different transport pathways may lead to a variety of δ^53^Cr_*M. edulis*_ values of between −0.20‰ and 0.58‰ and thus to variations in δ^53^Cr values of *Mytilus*. Variable Cr(III) and Cr(VI) concentrations in sea water are capable to create δ^53^Cr_*M. edulis*_ values similar to as well as lower than δ^53^Cr_sea water_ values, as it was observed in bulk *M. edulis* from Kiel Fjord.

### 4.2 Environmental factors causing Cr isotope fractionation prior to uptake

There can be a large variance in δ^53^Cr values from the same or proximate sampling sites. For example, *Mytilus* from Sejerø Bay (Zealand), Storebælt (Funen) and Kiel Fjord (DE) yield bulk δ^53^Cr values from −0.05 ± 0.08‰ to 1.25 ± 0.10‰. This indicates that single organisms respond to micro‐environmental conditions, which may then have a large impact on the δ^53^Cr value of a shell.

**Figure 9:**
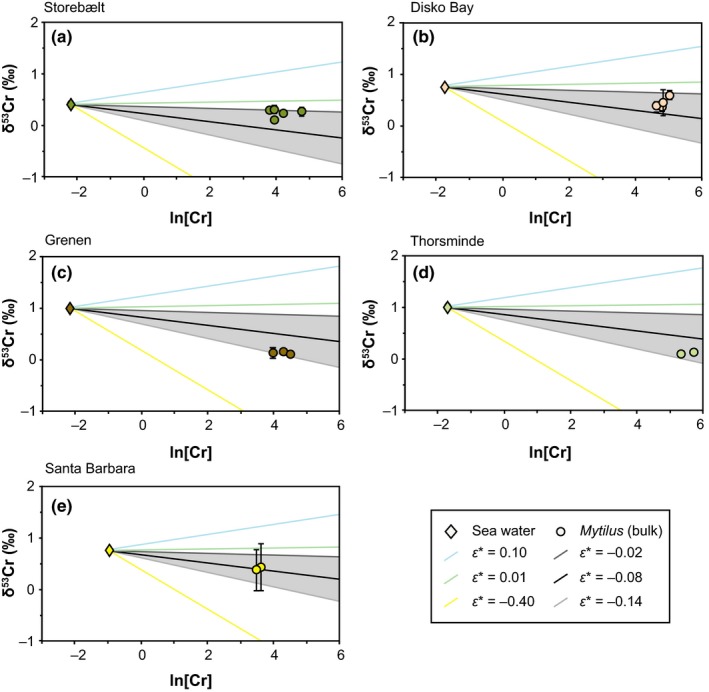
Ln[Cr] ([Cr] in ppb) and δ^53^Cr values of *Mytilus* (circle) from each location (a) Storebælt (Baltic Sea), (b) Disko Bay (North Atlantic), (c) Grenen (Skagerrak), (d) Thorsminde (North Sea), (e) Santa Barbara (North Pacific) are indicated by a different colour; ambient sea water samples are represented by diamonds. Lines indicate fractionation factors (ε*; *f* ≥ 0) of Cr reduction (yellow; Han et al., 2012) and Cr oxidation (green and blue; Zink et al., 2010; Bonnand et al., 2013) and black and grey lines (highlighted by grey‐shaded area) indicate ε* values that best represent the Cr fractionation of most *Mytilus edulis* samples (Kiel Fjord) [Colour figure can be viewed at wileyonlinelibrary.com]

Despite the possibly large range of δ^53^Cr values, average δ^53^Cr values of samples from individual sampling sites are distinct and statistically discernible (Figure 4; Table 4). Specimens collected in the Baltic Sea show similar average δ^53^Cr values (0.21 ± 0.08‰ to 0.23 ± 0.17‰). In addition, the ε* values of *Mytilus* shells can be grouped according to their location (Table 4). While *Mytilus* from Storebælt (DK) yield ε* ≈ −0.02‰ (Figure 9a), samples from Grenen (DK) show a lower fractionation between sea water and *Mytilus* (ε* = −0.14‰; Figure 9c), indicating a preferential uptake of isotopically light Cr. Thus, location‐specific environmental conditions such as salinity, OM or water depth that significantly impact δ^53^Cr_sea water_ values (e.g., Goring‐Harford et al., 2018; Paulukat et al., 2016; Scheiderich, Amini, Holmden, & Francois, 2015) also control the Cr isotope signature incorporated into *Mytilus* shells.

#### 4.2.1 Micro‐environmental sea water conditions

*Mytilus* are sessile and attach to a substrate (e.g., rock or older *Mytilus* shell) as larvae or juveniles, and younger individuals may overgrow older ones. Thus, small‐scale changes (e.g., mucus) in sea water chemistry may significantly influence the δ^53^Cr values of some *Mytilus* while others are unaffected. We hypothesise that in a dense population of *Mytilus*, a chemical gradient may establish between well‐mixed open sea water containing mainly dissolved Cr(VI) and restricted organic‐rich sea water (Cr(III)) caught in the limited space between shells. *Mytilus* growing on the periphery of the colony have access to “unused” sea water with low Cr(III) concentrations. Around the innermost individuals, OM from biodeposits (faeces and pseudofaeces, including mucus [protein‐polysaccharide complexes]) and microalgae may accumulate due to limited exchange with “unused” sea water under low energy conditions. The presence of mucus further stimulates the growth of microalgae (Cognie & Barikle, 1999). In the presence of large amounts of dissolved OM in the restricted central area of a colony, the solubility of Cr(III) may increase as it is known to be stabilised in solution by organic complexation (Sander & Koschinsky, 2000). We propose that increased OM concentrations stabilising Cr(III) in solution in restricted dense parts of the *Mytilus* colonies may induce Cr fractionation within sea water, potentially leading to heterogeneous [Cr] and isotopic compositions within a *Mytilus* colony (e.g., *M. edulis* from Kiel Fjord). However, since the position of individual shells relative to other shells was not recorded during our sampling campaign, we are unable to substantiate the above hypothesis.

#### 4.2.2 Local environmental sea water conditions

The different average δ^53^Cr values measured in *Mytilus* from Kiel Fjord, Disko Bay and Thorsminde could suggest that each sampled a local Cr pool of a water body that is isotopically distinct. *Mytilus* shell samples influenced by the low‐saline Baltic Sea have approximately 0.1‰ higher δ^53^Cr values than samples from the more saline North Atlantic coasts (Figure 4). The elevated δ^53^Cr value measured in *Mytilus* from Limfjord (DK) might be caused by restriction of the water body and its overall lower salinity (brackish water, 6 PSU; Lewis et al., 2013). Similarly, elevated δ^53^Cr values in two *M. edulis* from Kiel Fjord may be influenced by the strong annual variation in salinity 2016:(13–24 PSU; bsh.de). The δ^53^Cr_sea water_ values are influenced by the salinity of the water body (e.g., heavy rainfalls diluting the water bodies with freshwaters, elevated temperatures leading to increased salinities due to evaporation). Riedel (1984) found that high salt and therefore also high sulphate contents can reduce the uptake of Cr(VI) as it is in competition with chromate. Low salinity and high Cr(VI) conditions on the other hand facilitate the uptake of Cr(VI) by algae (Wang, Griscom, & Fisher, 1997) and therefore increase the δ^53^Cr values in the remaining sea water as discussed above. Changes in freshwater input can thus enhance the δ^53^Cr variability in mollusc shells. This fits well with our δ^53^Cr results around Denmark.

The isotopically light *Mytilus* from Syracuse (IT; −0.04 ± 0.26‰) may be explained by comparably higher primary productivity in the warmer Mediterranean Sea relative to northern ocean basins and therefore by an increased food availability associated with light Cr. The frequency of algal blooms in different water bodies may influence δ^53^Cr variability in biogenic carbonates as algae blooms can significantly affect δ^53^Cr values of sea water. For example, seasonal algae blooms in the Sound (DK) occur from late summer to autumn. These algae blooms may cause the variability in δ^53^Cr values between approximately 0.63 ± 0.13‰ in October and 0.35 ± 0.11‰ in June (Køge Bay) (Paulukat et al., 2016), as Cr was shown to be reduced to Cr(III) under high OM conditions (Semeniuk, Maldonado, & Jaccard, 2016; Sikora et al., 2008). The isotopically heavy sea water samples in October and April may be caused by algal reduction of Cr, where ^52^Cr(III) is preferentially removed from solution by algae. *Mytilus* from areas with high algae production may thus differ from samples from low‐productivity sites.

### 4.3 Scenarios leading to heterogeneous δ^53^Cr_*Mytilus*_ values

Connecting the observed Cr fractionation factors and δ^53^Cr variability to the Cr uptake model as well as to environmentally controlled sea water biogeochemistry, we can describe different scenarios for open and closed shell valves (open and closed systems) that are able to cause the observed δ^53^Cr variability. The periostraca are excluded from these scenarios as Cr associated with the organic outer sheath is likely taken up directly from sea water, similarly to Cr uptake into byssal threads (Chassard‐Bouchaud et al., 1989).

#### 4.3.1 Open system

The δ^53^Cr values similar to or higher than sea water with high Cr fractionation factors (ε* ≈ 0.01–0.10‰) are observed in few bulk shell samples from Kiel Fjord (DE‐KI_3: δ^53^Cr = 0.74 ± 0.25‰ and DE‐KI_19: δ^53^Cr = 1.25 ± 0.10‰). *Mytilus* specimens growing at the periphery of the colony may filter sea water with elevated δ^53^Cr_sea water_ values as they have access to dissolved Cr(VI) and are less exposed to increased Cr(III) concentrations compared with specimens growing in the OM‐ and Cr(III)‐rich central areas (Section 4.2.1). As no information on growth position of *Mytilus* samples relative to other specimens is available, we can only infer that they may have been growing on the periphery of a colony or in loosely populated subtidal areas with access to Cr(VI)‐rich sea water. Further, the Cr isotopic composition of sea water is heterogeneous (Scheiderich et al., 2015) and it is likely that the sea water samples from Kiel Fjord we analysed in this study do not represent the full range of δ^53^Cr values that may occur in Kiel Fjord. For example, increased reductive Cr removal in the presence of an algae bloom may lead to δ^53^Cr_sea water_ values exceeding the sea water values we measured (maximum δ^53^Cr_sea water_ value for Kiel Fjord analysed in this study: 0.62 ± 0.19‰). Provided an uptake mechanism that is capable of transporting this isotopically heavy Cr(VI) with little or no fractionation (e.g., via sulphate channels) from the body cavity to the EPS, biogenic carbonates can potentially record δ^53^Cr values that are elevated compared to average sea water values (Ia, Figure 8a).

The majority of *M. edulis* shell samples from the Kiel Fjord (ε*_average_ ≈ −0.08‰) and all samples from Grenen (DK, Skagerrak; ε* ≈ −0.14‰), Thorsminde (DK, North Sea; ε* ≈ −0.13‰) and Santa Barbara (United States, North Pacific; ε* ≈ −0.08‰; Figure 9a–e) show a negative offset between *Mytilus* shells and ambient sea water. This indicates that Cr(III) incorporation is more efficient than Cr(VI) incorporation in *Mytilus*. Variable concentrations of Cr(VI) (Figure 8, I) and Cr(III) (Figure 8, II) in sea water may significantly influence the δ^53^Cr values of biogenic carbonates and thus *Mytilus* (Wang et al., 2016). Low δ^53^Cr values were observed in *M. edulis* from Kiel Fjord (e.g., DE‐KI_42: −0.14 ± 0.14‰) compared with *Mytilus* from Santa Barbara (e.g., STA‐1: 0.45 ± 0.07‰). Assuming nearly 100% Cr(III) with δ^53^Cr = −0.12 ± 0.10‰ in sea water, δ^53^Cr_*M. edulis*_ values may become as low as −0.20‰ using a conservative $\varepsilon_{2}^{*}$ of −0.04‰ (II), Figure 8a). This indicates that uptake of isotopically light Cr(III) into *Mytilus* may be facilitated under high Cr(III) concentrations in sea water (Figure 8a), for example, in presence of mucus. Such conditions prevail in densely populated *Mytilus* colonies. Additionally, gaping shell valves may induce partial reduction of Cr(VI) to Cr(III) during shell valve closure (step 1, Figure 8a). Prolonged valve closure, for example, caused by low availability of food, may increase Cr isotope fractionation ($\varepsilon_{1}^{*}$ < −0.04‰) resulting in low δ^53^Cr_Mytilus_ values. Furthermore, Cr may be reduced in vesicles (step 2, Ib and II, Figure 8a,b) where it is associated with sulphur and phosphorus (Chassard‐Bouchaud et al., 1989).

#### 4.3.2 Closed system

*Mytilus* shell samples showing δ^53^Cr values close to sea water (ε* ≈ −0.02‰) were found at Storebælt (GL‐DB3: 0.57 ± 0.09‰), Disko Bay (DK‐Fyn2: 0.30 ± 0.07‰) as well as in Kiel Fjord (DE‐KI_49: 0.40 ± 0.08‰, DE‐KI_peeled_36: 0.37 ± 0.09‰, DE‐KI_NaOCl_52: 0.46 ± 0.05‰, DE‐KI_NaOCl_68: 0.37 ± 0.09‰). In a closed system (shell valves closed for up to several days; Babarro and Zwaan (2008)), Cr reduction in sea water trapped between the shell valves may be quantitative, leading to a Cr fractionation factor of $\varepsilon_{1}^{*}$ = 0‰ (Ib, Figure 8b). Step 2 may then induce presumably constant and small Cr isotope fractionation (e.g., $\varepsilon_{2}^{*}$ = −0.04‰), leading to δ^53^Cr_*M. edulis*_ values similar to or only slightly lower than the ambient sea water value (Ib and II, Figure 8b). A closed system with closed shell valves can occur during periods of starvation or due to exposure to air. *M. edulis* samples from Kiel Fjord are not subjected to significant tidal influence, but starvation periods are possible and may cause nearly quantitative Cr reduction.

### 4.4 Cr isotope offset between bulk *Mytilus* shells and ambient sea water

While offsets between δ^53^Cr values of shells and ambient sea water are relatively large (L_peeled_ approximately $\Delta^{53}{\text{Cr}}_{\mathrm{sea}\;\;\mathrm{water}}^{\mathrm{Lpeeled}}$ = −0.37‰, Table 3), the calculated δ^53^Cr values for the corresponding periostraca (based on our calculations in Equation (2): 0.37‰) closely resemble sea water values ($\Delta^{53}{\text{Cr}}_{\mathrm{sea}\;\;\mathrm{water}}^{\mathrm{periostracum}}$ = −0.19‰, Table 3). Using targeted leaches, we determined that up to 40% of the total Cr in a *Mytilus* shell is associated with the periostracum, suggesting that it is the primary Cr‐bearing phase in these samples. Further, based on mass balance arguments, we estimate an average δ^53^Cr composition for periostraca from our Kiel Fjord samples to be 0.37‰. These values are close to δ^53^Cr_sea water_ values measured at the same location, indicating that little fractionation occurred during Cr uptake into the periostracum. This suggests that Cr(VI) may adsorb directly from sea water and that uptake of Cr(VI) into the periostracum may be quantitative, leaving no time for fractionation processes to take place. This is in agreement with previous findings that Cr may be adsorbed onto byssal threads of *Mytilus* directly from sea water (Chassard‐Bouchaud et al., 1989).

The majority of the bulk *Mytilus* samples from different sampling sites measured in this study shows a negative offset from ambient sea water $\Delta^{53}{\text{Cr}}_{\mathrm{sea}\;\;\mathrm{water}}^{\mathrm{bulk}\;\;Mytilus}$ of between 0.17 and 0.91‰ (Table 3), despite the large variability in δ^53^Cr values (from −0.30 ± 0.11 to +1.25 ± 0.10‰ 2SD). This supports the preferential incorporation of lighter Cr into primary (biogenic) carbonates during precipitation (e.g., Pereira et al., 2015). All samples from the Baltic Sea lie within a small range of offsets, from 0.17‰ in Storebælt (δ^53^Cr_sea water_ from Paulukat et al. (2016)) to 0.33‰ in Kiel Fjord (DE). These similar $\Delta^{53}{\text{Cr}}_{\mathrm{sea}\;\;\mathrm{water}}^{\mathrm{bulk}\;\;Mytilus}$ values found in *Mytilus* of proximate locations indicate that average δ^53^Cr values are corresponding to local sea water redox chemistry.

The range of $\Delta^{53}{\text{Cr}}_{\mathrm{sea}\;\;\mathrm{water}}^{\mathrm{carbonate}}$ values found in this study is in agreement with previous results, revealing a consistently negative offset between δ^53^Cr values of (mostly biogenic) carbonates and their ambient sea water ($\Delta^{53}{\text{Cr}}_{\mathrm{sea}\;\;\mathrm{water}}^{\mathrm{carbonate}}$; Figure 5; Bonnand et al., 2013; Pereira et al., 2015; Wang et al., 2016; Farkaš et al., 2018; Frei et al., 2018). These offsets range from 0.00 to 0.35‰ in ooids (Bonnand et al., 2013), ≈0.3 to 0.4‰ in modern bivalves (Frei et al., 2018), ≈0.45‰ in modern marine biogenic carbonates from the Great Barrier Reef (Farkaš et al., 2018), 0.46 ± 0.14‰ (2SD) in modern marine carbonate sediments from the Caribbean Sea (Holmden et al., 2016) to approximately 0.9‰ in corals (Pereira et al., 2015).

## 5 CONCLUSIONS

The heterogeneity of δ^53^Cr values of analysed shell fractions of *M. edulis* from Kiel Fjord and bulk *Mytilus* shells from a range of other sample sites (−0.30 ± 0.11‰ to +1.25 ± 0.10‰ 2SD) indicates that besides uptake of Cr(III) as suggested by previous studies (e.g., Frei et al., 2018; Pereira et al., 2015; Wang et al., 2016), significant amounts of Cr(VI) can be incorporated into *Mytilus* shells. We hypothesise that the heterogeneous δ^53^Cr values are mainly controlled by i) vital effects (shell valve closure time), ii) micro‐environmental parameters such as the presence of mucus that influence shell valve closure time and thus the extent of Cr reduction and iii) sea water biogeochemistry of the local environment (e.g., salinity) influencing the local δ^53^Cr_sea water_ value. Our model for Cr incorporation into *Mytilus* is compatible with the heterogeneity of δ^53^Cr values in the analysed *Mytilus* shells and is capable of explaining the mostly negative Cr isotopic offset between *Mytilus* shells and sea water that may be induced by Cr reduction during shell valve closure time and during cellular Cr transport. The feeding mechanism of *Mytilus* involves frequent closure of the shell valves, which is likely an important control on δ^53^Cr values. During shell valve closure, anoxic conditions immediately evolve in the sea water trapped between the valves and Cr may be partially or quantitatively reduced. Thus, Cr reduction likely takes place in the sea water trapped between the closed shell valves. Cr(III) and Cr(VI) contained in sea water that is either filtered with open shell valves (open system) or trapped between closed shell valves (closed system) can be vacuolised by epithelial cells and reach the EPS via exocytosis, perhaps including Cr reduction to insoluble Cr(III). As an alternative pathway, Cr(VI) can be transported using sulphate channels. We found evidence that only small proportions of Cr (≤3.4 ppb) are incorporated in the crystal lattice of carbonate minerals. Instead, the shell organic matrix may be the main Cr‐hosting fraction and incorporate both Cr(VI) and Cr(III) adsorbed onto organic macromolecules such as ligands or sulphated polysaccharides. Additionally, the periostracum may host significant proportions of Cr (up to 534 ppb) and Cr may directly diffuse from sea water into or adsorb onto the periostracum.

Sea water biogeochemistry may further influence the pronounced heterogeneity of δ^53^Cr values in *Mytilus* shells from both within a colony and from different locations. Micro‐ (e.g., gradient of mucus and thus OM from central to peripheral colony) and regional environmental conditions (e.g., algae blooms, salinity) impact δ^53^Cr_sea water_ values and thus the Cr signature incorporated by *Mytilus*.

The resulting offsets $\Delta^{53}{\text{Cr}}_{\mathrm{sea}\;\;\mathrm{water}}^{\mathrm{bulk}\;\;Mytilus}$ range from 0.17 to 0.91‰ and are consistent with previously reported offsets of biogenic carbonates. *Mytilus* from two locations around the Baltic Sea (Kiel Fjord and Storebælt) show similar $\Delta^{53}{\text{Cr}}_{\mathrm{sea}\;\;\mathrm{water}}^{\mathrm{bulk}\;\;Mytilus}$ values; however, δ^53^Cr variability is high in both sea water and *Mytilus* and we do not fully understand the processes inducing this variability yet. Before Cr isotopes in fossil *Mytilus* can be used to reliably reconstruct past redox changes, a detailed understanding of processes fractionating Cr during and prior to Cr uptake and investigations on the preservation potential of Cr signatures in organic matrices of molluscs are crucial.
